# Implementing Key Drivers for Diabetes Self-Management Education and Support Programs: Early Outcomes, Activities, Facilitators, and Barriers

**DOI:** 10.5888/pcd15.170399

**Published:** 2018-01-25

**Authors:** Jennifer Murphy Morgan, Yvonne Mensa-Wilmot, Shelly-Ann Bowen, Monica Murphy, Timethia Bonner, Stephanie Rutledge, Gia Rutledge

**Affiliations:** 1 Division of Diabetes Translation, National Center for Chronic Disease Prevention and Health Promotion, Centers for Disease Control and Prevention, Atlanta, Georgia; 2 Health, Research, Informatics and Technology: Public Health Division, ICF International, Atlanta, Georgia; 3 Oak Ridge Institute for Science and Education, Atlanta, Georgia

Diabetes, a serious and costly condition, is characterized by illness and death from long-term microvascular and macrovascular complications (1). Additionally, numerous and well-known comorbidities can accompany diabetes, including cardiovascular disease, retinopathy, amputations, and nephropathy (1). Often these complications and comorbidities interfere with a person’s ability to self-manage their diabetes (2). The Centers for Disease Control and Prevention (CDC) projects that as many as 1 in 3 adults could have diabetes by 2050 (3). In 2012, the United States spent an estimated $245 billion on diabetes care, including $176 billion in direct medical costs and $69 billion in indirect costs from lost workdays, restricted activity, disability, and early death (4). Many costly complications among people with diabetes can be prevented or delayed with appropriate preventive care and self-management (5).

CDC’s National Center for Chronic Disease Prevention and Health Promotion leads efforts to address the chronic disease burden effectively and equitably in the US population. Generally positioned as the primary public health authority supporting the delivery of public health services within a state, the state health department is a unique partner for the collaborative implementation of population-focused interventions. The 5-year cooperative agreement SPHA1305 (State Public Health Actions to Prevent and Control Diabetes, Heart Disease, Obesity and Associated Risk Factors and Promote School Health) is such a partnership, involving 4 CDC divisions, all 50 state health departments, and the District of Columbia, to develop strategies to reduce the risk factors for obesity and the management and prevention of chronic conditions such as type 2 diabetes. Through this partnership, CDC’s Division of Diabetes Translation provides scientific leadership and technical expertise to support implementation of cross-cutting approaches to improve diabetes outcomes nationally. This essay reflects on the first 3 years of activity of the cooperative agreement.

## Clinical and Community Linkages To Support Diabetes Self-Management

One way to improve diabetes management is to increase linkages between community resources and clinical services. Diabetes self-management education and support (DSMES) programs connect people with diabetes to effective clinical services in their communities. DSMES is usually offered to patients at diagnosis, during annual assessments, and when transitions or new disease complications occur that influence self-management and is guided by evidence-based standards. DSMES is an individualized process in which health care providers incorporate information on the needs, goals, and life experiences of patients when imparting knowledge, teaching skills, and coaching for behavioral change necessary for diabetes self-care (6). Through an assessment of program structure, process, and outcomes, the American Diabetes Association (ADA) and the American Association of Diabetes Educators (AADE) recognize or accredit organizations providing DSMES programs to assure quality.

Studies show that participants in DSMES programs reduce glycosylated hemoglobin (HbA1c) levels, have fewer emergency department visits, and incur lower in-patient costs (7). Findings of a longitudinal study over a 10-year period showed that each 1% reduction in HbA1c was associated with reductions in risk of 21% for diabetes-related deaths, 14% for myocardial infarctions, and 37% for microvascular complications (8). Significant decreases in in-patient costs, a primary source of savings for Medicaid and commercial payers, have been attributed to DSMES (9).

## Assessing Key Activities Implemented by State Health Departments

Increasing the number of DSMES programs in communities and securing Medicaid reimbursement in states with no DSMES coverage for beneficiaries are critical goals of cooperative agreement SPHA1305. State health departments partner with health systems and community organizations to increase DSMES program access, patient referrals, and reimbursement. The partners’ activities are anchored in 4 promising practice areas known to drive implementation: 1) supporting organizations in establishing ADA-recognized or AADE-accredited DSMES programs, 2) securing Medicaid coverage for DSMES, 3) establishing referral policies and practices in health care systems to efficiently connect people to DSMES programs, and 4) raising awareness and enhancing the capacity of people with diabetes to participate in DSMES. Numerous state health departments have implemented such activities (Table 1). 

**Table 1:** Examples of Diabetes Self-Management Education and Support Activities Implemented by State Health Departments, 2012–2014

Strategy Driver	Example Activities
ADA-recognized or AADE-accredited DSMES programs established (primary or satellite sites)	The Alabama Department of Public Health created an advisory group to work with department staff members to identify existing DSMES programs, areas of the state underserved or unserved by DSMES programs, and organizations interested in becoming providers of accredited or recognized programs, and to determine which strategies should be pursued in which areas to increase access and referrals to and use of DSMES programs.
	Arizona Department of Health Services staff members provided technical assistance and training to 3 organizations in Arizona to obtain AADE accreditation (eg, capacity building within each organization, curriculum development, credentialing compliance, training of staff on evidence-based strategies).
Insurance coverage for DSMES	The Illinois Department of Public Health engaged with Medicare/Medicaid Alignment Initiative Health Plans to discuss DSMES coverage for patients/members with diabetes.
	Staff members of the Indiana State Department of Health provided live webinars on DSMES reimbursement for 20 hospital-based and 4 pharmacy-based DSMES programs.
Referral policies and practices in place in the health system to efficiently connect people with diabetes to DSMES programs	Members of the Nevada Diabetes Education Stakeholder group, created by the Nevada Department of Health and Human Services, used a DSMES academic detailing toolkit to educate health care providers on ways to increase self-care options for patients and make referrals to ADA-recognized or AADE-accredited DSMES programs.
	Maryland Department of Health and Mental Hygiene staff members designed and built an online self-management referral website that allows the public to search for DSMES classes and health care providers to refer patients to DSMES programs.
Awareness, capacity, and willingness of people with diabetes to attend DSMES programs when other drivers are in place	Michigan Department of Health and Human Services staff members expanded media promotion of recognized or accredited DSMES programs through their diabetes program website and a statewide radio public service announcement.
	The New York State Department of Health’s diabetes program partnered with the state’s arthritis program to develop a digital media campaign to promote DSMES among women aged 40 or older in 2 counties.

Abbreviations: ADA, American Diabetes Association; AADE, American Association of Diabetes Educators; DSMES, diabetes self-management education and support.

Assessment of program activities to monitor and understand how the activities lead to improved health outcomes is critical to the success of any system-wide intervention. Performance monitoring provides useful and timely information on strengths and opportunities for improvement and on how to tailor technical assistance for midcourse corrections.

In year 3 of the 5-year cooperative agreement, we examined data on the progress made by analyzing the annual reports of the 51 grant recipients. We abstracted such data as quantitative performance measures describing the reach of activities, the number of ADA-recognized and AADE accredited DSMES programs, the proportion of counties with ADA-recognized and AADE accredited DSMES programs, the number of Medicaid recipients with DSMES as a covered Medicaid benefit, and the number of people with at least one encounter at an ADA-recognized or AADE-accredited DSMES program. Overall, 43 states were implementing activities to address DSMES access, participation, and/or coverage. Our analysis included data reported only by state health departments that provided data for a given measure for all 3 years: 2012, 2013, and 2014 (Table 2). The proportion of counties offering DSMES programs increased from 54.7% at baseline to 57.0% in year 3 (based on data from 38 states). The overall number of DSMES programs increased by 7.8% from 2,822 to 3,043 (based on data from 41 states). We also found a 12.6% increase in the number of Medicaid beneficiaries with DSMES as a covered benefit, from 1.26 million to 1.42 million (based on data from 20 states). The number of people with diabetes who had at least 1 session at an ADA-recognized or AADE-accredited DSMES program went up by 16.6%, from 906,402 at baseline to 1,057,194 by year 3 (based on data from 50 states and the District of Columbia) (Table 2).

**Table 2:** Performance Measures for Diabetes Self-Management Education and Support (DSMES) Activities Implemented by State Health Departments^a^

Funding Year	No. of ADA-Recognized and/or AADE-Accredited DSMES Programs	Proportion of Counties with ADA-Recognized and/or AADE-Accredited DSMES Programs	No. of Medicaid Recipients With DSMES as a Covered Medicaid Benefit	No. of People With ≥1 Encounter at an ADA-Recognized and/or AADE-Accredited DSMES Program
No. of state health departments reporting data for all 3 years	41	38	20	51
Baseline (2012)	2,822	54.7	1,258,042	906,402
Year 2 (2013)	3,117	57.8	1,204,677	1,049,473
Year 3 (2014)	3,043	57.0	1,417,124	1,057,194
Percentage change from 2012 to 2014	7.8	4.2	12.6	16.6

ADA, American Diabetes Association; AADE, American Association of Diabetes Educators; DSMES, diabetes self-management education and support.

a Analysis included data reported only by state health departments that provided data for a given category for all 3 years (2012, 2013, and 2014).

## Supportive Partnerships in Diabetes Self-Management Education and Support

Analysis of information in the annual reports on the particular activities of 43 state health departments that implemented DSMES-related activities were coded according to the 4 promising practice areas known to drive implementation. In addition, barriers and facilitators reported by 16 state health departments that elected to evaluate their progress were analyzed.

Health departments and their partners undertook a wide range of activities. They worked to expand program locations to worksites and faith-based organizations; convened advisory groups to identify existing programs interested in obtaining ADA-recognition or AADE-accreditation; sponsored diabetes symposia to provide education for clinical staff, pharmacists, payers, and interested stakeholders on appropriate billing and coding for DSMES services, sustainability strategies, and reimbursement models; and worked with partners to survey health care providers to increase referrals to DSMES programs. Some state health departments developed data sharing agreements to automate DSMES program referrals through electronic health records, while others developed toolkits and educational materials for health care providers. Some developed radio public service announcements and engaged community health workers to raise awareness and increase program participation in the community. Additionally, several states posted maps of DSMES program locations on websites. Health departments entered into partnerships with Federally Qualified Health Centers (FQHCs), medical practices, diabetes coalitions, and pharmacists to advocate for adoption and sustainability of DSMES programs and provided technical assistance to programs seeking AADE accreditation or ADA recognition. 

Health departments reported that the inclusion of DSMES as a preventive service in the state’s Medicaid expansion program was critical to success. Establishing champions and creating advocacy for policy change through statewide diabetes coalitions were also vital. Having similar software for electronic health records across FQHCs, using a statewide database of health information resources and programs, and having health care providers who were willing to refer patients to programs increased patient participation. DSMES programs that held classes in easily accessible locations and at convenient times and that used culturally and linguistically appropriate curricula increased participation rates.

Challenges that affected program availability and access included the application process for AADE accreditation and ADA recognition. Further analysis showed that state health departments have limited staff to support the processes of accreditation, recognition, and compliance. Other challenges were a lack of site-level assessment data on DSMES programs; clinicians’ concerns about low insurance reimbursement rates, not getting reimbursed, and complicated reimbursement processes; scheduling, transportation, and child care difficulties; and limited availability of culturally and linguistically appropriate programs.

## Future Directions

Assessment of the progress made in implementing DSMES programs under cooperative agreement SPHA1305 provides information to develop guidance for helping state health departments identify how to further improve results by the end of the SPHA1305 funding cycle. In addition, information on barriers and facilitators will inform and guide technical assistance and training provided by the Division of Diabetes Translation for the remainder of the cooperative agreement. The Division of Diabetes Translation developed a series of interactive webinars to build the evaluation capacity and enhance completeness and quality in data reporting. Topics included improving data quality along with developing and disseminating health impact statements and program success stories to various audiences. Continued attention to program activities and performance monitoring data with a goal of real-time action to overcome challenges and provide technical assistance will ensure that our partners promote sustainable strategies for improved health outcomes in diabetes management.
